# Effectiveness of mechanical traction as a non-surgical treatment for carpal tunnel syndrome compared to care as usual: study protocol for a randomized controlled trial

**DOI:** 10.1186/1745-6215-15-180

**Published:** 2014-05-22

**Authors:** Margreet Meems, Brenda Den Oudsten, Berend-Jan Meems, Victor Pop

**Affiliations:** 1 Department of Medical and Clinical Psychology, Center of Research on Psychology in Somatic diseases (CoRPS), Tilburg University, PO Box 90153, 5000, LE Tilburg, Netherlands; 2 Department of Education and Research, St. Elisabeth Hospital, PO Box 9015 5000 LE Tilburg Netherlands; 3 Department of Neurology, VieCuri Medical Center, PO Box, 1926, 5900 BX Venlo, Netherlands

**Keywords:** Carpal tunnel syndrome, Mechanical traction, Phystrac, Boston Carpal Tunnel Questionnaire

## Abstract

**Background:**

Carpal tunnel syndrome (CTS) is a common condition (prevalence of 4%) where the median nerve is compressed within the carpal tunnel resulting in numbness, tingling, and pain in the hand. Current non-surgical treatment options (oral medication, corticosteroid injections, splinting, exercise, and mobilization) show limited effects, especially in the long-term. Carpal tunnel release (CTR) surgery is effective in 70 to 75% of patients, but is relatively invasive and can be accompanied by complications. In an observational study, mechanical traction proved to be effective in up to 70% of patients directly after treatment and in 60% after two years follow-up. This randomized controlled trial (RCT) will examine the effectiveness of mechanical traction compared to care as usual in CTS.

**Methods/Design:**

Patients diagnosed with CTS will be recruited from an outpatient neurology clinic and randomly assigned to the intervention group (mechanical traction) or the control group (care as usual). Participants in the intervention group will receive 12 treatments with mechanical traction during six consecutive weeks. Primary outcome is symptom severity and functional status, which are measured with the Boston Carpel Tunnel Questionnaire (BCTQ). Secondary outcomes are quality of life (WHOQOL-BREF), health related resource utilization, and absenteeism from work. Outcomes will be assessed at baseline, and at 3, 6, and 12 months after inclusion. Linear mixed effect models will be used to determine the change from baseline at 12 months on the BCTQ, WHOQOL-BREF, absenteeism from work and health related resource utilization. The baseline measurement, change from baseline at three and six months, as well as duration of symptoms until inclusion, age, gender, and co-morbidity will be included as covariates The Pearson’s correlation coefficient will be generated to assess the correlation between depression and anxiety and treatment outcome.

**Discussion:**

Since current non-surgical treatment options are not effective long-term and CTR is relatively invasive, there is a need for an effective and non-invasive treatment option. Mechanical traction is a safe treatment option that may provide a good alternative for the usual care. Considering the prevalence of CTS, the study is of great clinical value to a large patient population.

**Trial registration:**

Clinical Trials NL44692.008.13 (registered on 19 September 2013).

## Background

Carpal tunnel syndrome (CTS) is a common compressive neuropathy in which the median nerve is compressed at the level of the carpal tunnel [1,2]. CTS can occur in one or both hands, and is either idiopathic (spontaneous) or dynamic (only during certain movements). The compression leads to numbness and tingling in the first three fingers and the radial side of the ring finger, paresthesia, pain, and (in severe cases) weakness. The symptoms are often worse at night or after use of the hand [2]. Prevalence is estimated to be about 4% in the general population [3] and up to 10% in the working population [4]. Although CTS can occur at any age, it most commonly occurs between 40 to 60 years and its prevalence is higher in women compared to men [1]. Obesity, diabetes mellitus, and alcohol abuse are risk factors for developing CTS [1]. Occupation has been identified as an environmental risk factor: vibration, hand force, and repetition are associated with increased risk for developing CTS [5]. Furthermore, there is a psychological component to the experience of CTS symptoms: depression has been reported as a predictor of pain in CTS patients [6].

CTS can be diagnosed using electromyography (EMG). Compression of the median nerve can lead to damage and dysfunction of the myelin sheath, resulting in slowed conduction velocity, which can be detected using EMG [7]. Treatment options to reduce the compression and relieve symptoms can be roughly divided into surgical and non-surgical procedures. Non-surgical, less invasive treatment options are numerous, including oral medication, corticosteroid injections, splinting, exercise, and mobilization interventions [8-12]. However, there is only short-term or limited evidence of benefit for these interventions. Many (non-steroidal) drugs did not prove to be significantly superior compared to placebo [12]. There is only limited evidence for the effectiveness of splinting, exercise, and mobilization interventions [10,11]. Local corticosteroid injections provide considerable symptom relief and therefore show the best results of the non-surgical treatments [8,9]. However, corticosteroids seem to merely suppress CTS symptoms and the positive effects do not last [8,13]. The treatment effect diminishes over time and half of patients who receive corticosteroid injections experience recurrence of symptoms within a year [13].

Compared to non-surgical treatment, surgery is the only known treatment option that shows long-term positive effects [14]. The principle of the procedure, called carpal tunnel release (CTR), is to decompress the nerve by dividing the transverse carpal ligament [15]. Evidence suggests that CTR is a more effective treatment for CTS than splinting or oral medication, especially long-term [15]. However, up to 30% of patients who underwent CTR experience persistence or recurrence of CTS symptoms in the long-term or suffer from complications [16,17]. Therefore, there is still a clear need for an alternative non-invasive therapy, possibly making surgery redundant for a sub-category of patients.

Another promising non-surgical treatment for CTS is mechanical wrist traction using the Phystrac traction device. The Phystrac applies repeated traction movements to the wrist in different positions using gravitational force. Brunarski *et al*. described four case studies using mechanical traction that showed promising results [18]. In an observational study, physical therapists reported a success rate of 70% with mechanical traction immediately post-treatment [19], and 60% after two years follow-up [20]. However, until now, no randomized controlled trial (RCT) has been performed to show clinical evidence for the effectiveness of mechanical traction as compared to care as usual (surgical and non-surgical intervention).

### Hypothesis and objectives

The purpose of this study is to evaluate the effectiveness of mechanical traction in alleviating symptoms and improving hand function in patients with CTS compared to care as usual. The primary outcome is the change from baseline in symptom severity and functional status at 12 months, which is measured using the Boston Carpal Tunnel Questionnaire (BCTQ). Change from baseline in functional status and symptom severity at three and six months will be used as covariates. As secondary outcomes, we will assess quality of life, health related resource utilization, and absenteeism from work. Tertiary outcome is the impact of psychological distress on treatment outcome. We hypothesize that hand function and symptom severity will significantly improve more in CTS patients receiving 12 treatments with mechanical traction compared to CTS patients who receive care as usual after 12 months. Moreover, 12 treatments with mechanical traction will result in less absenteeism from work, a higher quality of life, and less health related resource utilization compared to care as usual in CTS patients after 12 months follow-up. Furthermore, a higher degree of depression and anxiety at baseline will result in a lower treatment effect in CTS patients.

## Methods/Design

### Study design

This study is designed as an RCT. Eligible patients diagnosed with CTS will be recruited from the outpatient neurology clinic of VieCuri Medical Center in Venlo, The Netherlands. They will be randomly allocated to the intervention (mechanical traction) or control (care as usual) group. The intervention group will receive 12 treatments with mechanical traction using the Phystrac traction device. The control group will receive care as usual, which may involve an expectant strategy, splinting, drug therapy, local corticosteroid injections or CTR.

### Eligibility

Adult men and women (aged 18 to 80 years) who have been diagnosed with CTS by means of a positive EMG will be invited to participate in our study. They have to be physically able to visit the outpatient clinic twice a week and sit in an upright position for at least 20 minutes. Patients with a previous history of CTS surgery will be excluded as well as those with insufficient understanding of the Dutch language. In addition, patients who have been diagnosed with another known (rare) cause of neuropathy, or who are suffering from a severe psychiatric disorder, such as personality disorder, schizophrenia or bipolar disorder will also be excluded.

### Recruitment, screening process and enrollment

Neurologists will select eligible patients based on the in- and exclusion criteria. During the visit to the outpatient clinic, the neurologist will inform patients about the study. In addition, the patients will receive an information letter. Two weeks after their visit to the outpatient clinic, they will be phoned by the researcher and asked whether they are interested to participate in the study. Eligible patients who are willing to participate will be invited for an intake visit at the hospital. During this intake, the eligibility of the patients will be double-checked and there will be an opportunity for the patients to ask additional questions about the study. Full written informed consent will be obtained from each participating patient. Subsequently, the patients will receive an interview and complete a set of questionnaires, which will result in the baseline measurement.

#### Ethical approval

The study protocol was approved by the Medical Ethical Committee of the St. Elisabeth Hospital in Tilburg, The Netherlands in August 2013 (reference number P1340). The RCT has been registered under the trial number: NL44692.008.13.

### Randomization

After inclusion, every patient will be randomly assigned to either the intervention or the care as usual group. Before the start of the study, a list of 200 random numbers of 1 (intervention) or 2 (care as usual) will be created using SPSS, version 21 (SPSS Inc,. Chicago, IL, USA). The random numbers will be uniformly distributed. The list will be kept at the secretary’s office of the outpatient neurology clinic. At the end of the intake, a participant number will be assigned to the patient. The secretary will refer to the generated random list and inform the researcher to which group the patient is allocated. The random list cannot be edited and is concealed from the researcher. The above described process of randomization will ensure objectivity of the researcher and will eliminate bias in the participants’ group allocation.

### Blinding

Blinding of patients or practitioners is not possible due to the current study design. As described previously, group allocation will not be known to both the patient and researcher during the intake. This allows for an objective baseline measurement.

### Intervention: Phystrac mechanical traction therapy

Patients in the intervention group will receive 12 treatments (twice a week for a period of six weeks) with the Phystrac mechanical traction device (type GR 10). The Phystrac provides mechanical traction to the wrist using weights between 1 and 18 kg. One treatment takes 10 to 15 minutes per diseased hand. The patient will be seated beside the traction device on a seat that is adjustable in height. The patient will put his arm on the arm support of the device and will be secured with two Velcro straps; one above and one below the elbow. Another Velcro strap which is attached to the weights will be fastened around the wrist with the palm of the hand upwards. The weight is set at 5 kg for women and 7 kg for men during the first treatment session. Every following treatment session, the weight is increased by 1 kg for women and 2 kg for men until 10 kg for women or 13 kg for men, or until the mechanical traction becomes painful or uncomfortable for the patient. When the patient is fitted correctly, the weight will be lowered and provides a pulling force on the wrist. After eight seconds the weight is lifted, providing a rest period of four seconds. This cycle will be repeated ten times. After 10 traction movements, the device stops and the researcher will rotate the wrist straps 30 degrees supination, after which another 10 traction movements will be provided. After that, a third set of 10 traction movements will be provided in 30 degrees pronation position. In total, 30 traction movements will be applied during each treatment. During the six weeks of treatment, patients will not receive other forms of treatment. After the six weeks of treatment, patients are allowed to receive usual CTS care when mechanical traction was not sufficient.

### Control group: ‘care as usual’

The control group will receive ‘care as usual’, which means they will receive regular treatment from their usual health care provider. Patients may adopt an expectant approach or receive treatment in the form of a wrist splint, local corticosteroid injections or CTR. Forms of treatment received in both groups will be documented during the full length of the study using questionnaires and medical records.

### Outcome measurements

Measurement time points and questionnaires used are shown in Table 1. Data collection will take place at baseline (before the start of the intervention) and at 3, 6, and 12 months after baseline in both groups. Additional data of the intervention group will be collected at two other time points: at 3 weeks (after 6 treatments) and at 6 weeks (after 12 treatments = immediately post-treatment). Information about patient attendance and drop-out will be recorded continuously. Patients of both groups will receive the follow-up questionnaires at 3, 6 and 12 months via Internet or a paper-and-pencil version with an addressed return envelope, depending on the participant’s preference.

**Table 1:** Measurements and time points

		**Measurement time points**					
**Measure**	**Questionnaire**	**T0**	**V1**	**V2**	**T1**	**T2**	**T3**
Patients’ background (demographics, clinical)	-	X			X	X	X
CTS symptom severity and functional status	BCTQ	X	X	X	X	X	X
Quality of life	WHOQOL-BREF	X		X	X	X	X
Depression and anxiety	PHQ-4	X	X	X	X	X	X
Health related resource utilization	Non-standardized	X					X
Absenteeism from work	Non-standardized	X			X	X	X

T0 = baseline, V1 = 3 weeks (only intervention group), V2 = 6 weeks (only invention group), T1 = 3 months, T2 = 6 months, T3 = 12 months, BCTQ = Boston Carpal Tunnel Questionnaire, WHOQOL-BREF = World Health Organization Quality of Life questionnaire abbreviated version, PHQ-4 = Patient Health Questionnaire-4.

### Primary outcome measure

#### Functional status and symptom severity

Self-reported functional status and symptom severity will be measured using the BCTQ [21,22]. The BCTQ is a disease-specific questionnaire referring to a typical 24-hour period in the past two weeks. It consists of two different scales: the Symptom Severity Scale (SSS) and the Functional Status Scale (FSS). The SSS comprises 11 questions about symptom severity, while the FSS consists of 8 daily activities which are rated based on degree of difficulty. The SSS and the FSS will be rated on a five-point scale. Both scales result in mean scores between 1 and 5, where greater impairment is represented by higher scores. The BCTQ is responsive to clinically relevant change and therefore an appropriate measure for treatment outcome [21]. It has been validated and is used in multiple studies to assess improvement in CTS symptoms over time [14], and also in The Netherlands [13,23].

### Secondary outcome measures

#### Quality of life

As a secondary outcome measure, self-reported quality of life will be measured using the abbreviated Dutch version of the World Health Organization Quality of Life (WHOQOL-BREF) [24,25]. The WHOQOL-BREF measures quality of life in four domains: physical health, psychological health, social relationships, and environment. In addition, it includes one facet on overall quality of life and general health. The WHOQOL-BREF consists of 26 items referring to the past two weeks, and which can be scored on a five-point scale, where a higher score represents a better quality of life. The WHOQOL-BREF has been proven to be a valid and reliable instrument [24,25].

#### Absenteeism from work

The number of days off work of each patient will be collected using a non-standardized questionnaire. Furthermore, participants will be asked whether they are on sick leave or have been because of their CTS complaints.

#### Health care related resource utilization

Patients will be asked how many times they visited a professional caregiver (general practitioner, physiotherapist, psychologist, specialist, or other health care providers) in the past 12 months, if they spent time in the hospital or used medication via a non-standardized questionnaire.

### Tertiary outcome measures

#### Depression and anxiety

The four-item Patient Health Questionnaire (PHQ-4) will be used to measure self-reported depression and anxiety. The questionnaire consists of two items on depression and two on anxiety, referring to the past two weeks. A higher score represents a higher level of anxiety and depression. The PHQ-4 is a reliable instrument that has been validated in a general population [26].

#### Demographic and clinical variables

Demographic variables will be collected during the intake interview. Information about age, education, job status, nationality, residence, and marital status will be documented. Also, BMI and life style habits (smoking, alcohol intake, physical activity) will be documented at baseline. Finally, clinical variables including medication and the existence of co-morbidity (for example, diabetes, cardiovascular disease, COPD) will be verified from the patient’s medical record forms after obtaining written informed consent.

## Statistical analysis

### Sample size and power calculation

The sample size calculation is based on a clinically relevant improvement from baseline on the functional and symptom severity scores of the BCTQ after 12 months follow-up. Sixty-four patients need to be included per treatment arm to statistically detect a minimum effect size of *d* = 0.5 between mean BCTQ scores of both groups with a power of 0.8 and a two-sided alpha of 0.05. A total of 200 patients will be included (100 patients per treatment arm), taking into account possible drop-out.

The following outcomes are defined. A completer is a patient who participated in at least 70% of the intervention sessions and the assessments. A responder is a patient who will have a reduction of 0.74 of the mean score (minimal clinical important difference) on the BCTQ compared to baseline [21]. A drop-out is a patient with less than 70% of the intervention or the follow-up data.

#### Planned analyses

The baseline characteristics of those who complete and will drop out during follow-up will be compared by means of an independent *t*-test for continues data and by Chi square analysis for categorical data. All analyses will be based on the intention-to-treat principle. Linear mixed effect models will be used to compare the change from baseline at 12 months between groups on the BCTQ, WHOQOL-BREF, absenteeism from work and health related resource utilization. Linear mixed effect models are able to adjust for missing values and are therefore used to avoid loss of information [27]. The baseline, three and six months measurements, as well as duration of symptoms until inclusion, age, gender, and co-morbidity will be included as covariates. Taking these covariates into account will adjust for differences within the groups and decrease variance. It will also adjust for possible baseline differences between groups [28]. When the data are normally distributed, Pearson’s correlation coefficients will be generated to examine the relationship between treatment outcome and depression and anxiety.

## Discussion

This paper describes the design for an RCT with the purpose to study the effectiveness of mechanical traction as a treatment for CTS compared to care as usual. Recruitment started in October 2013 in the VieCuri Medical Center in Venlo, The Netherlands. The outpatient neurology clinic registers over 400 CTS patients annually of whom about 350 will be eligible. During a period of 12 months, these patients will be invited to participate in the RCT. For several reasons, it is realistic to predict a response rate of 60%. First, the new treatment is painless and non-invasive. Second, in case of a non-responder, the patient can always make a choice for surgery without any evidence for poorer prognosis. The predicted sample size of 200 patients will easily enable the researchers in one year to include sufficient patients into the trial with sufficient power. The clinical relevance of this RCT is substantial. First, CTS is very common, not only in the general population (up to 4%), but especially in the working population (up to 10%). Secondly, because the prevalence in the working population is relatively high, CTS-related days of sick leave and workers’ compensation lead to an economic burden. Absenteeism from work after carpal tunnel release (CTR) is on average two to seven weeks [29,30]. In the US, cumulative excess loss of earnings of 4,443 workers who filed a CTS related workers’ compensation claim was estimated at $197 to $382 million over 6 years [31]. Thirdly, the long-term benefits of current treatment strategies of CTS are far from optimal. Fourth, it is well known that recovery from pain symptoms can be mediated by the patient’s mental state, especially anxiety and depression. Pain is a process from nociceptive registration to a subjective experience, which can be influenced by psychological wellbeing [32]. Depression has been reported as a predictor of pain intensity in CTS patients [6]. Research into the mediator effect of emotional distress on the outcome of CTS treatment is limited. Hobby *et al*. reported a significant association between BCTQ scores and scores on the depression and anxiety scales [33]. Moreover, Lozano Calderon *et al*. reported that patient dissatisfaction and perceived impairment after CTR can partly be predicted by depression [34]. Up until now, non-invasive CTS treatment mostly consists of splint therapy and corticosteroid injections. These treatments have not been proven to be effective in the long-term [8-10]. Invasive CTR, on the other hand, results in a positive outcome in only 70 to 75% of the patients in the long-term [16,17]. Mechanical traction using the Phystrac traction device is a promising treatment option. It is non-invasive and has been reported to result in substantial symptom relief in 70% of the patients, and in 60% of the patients two years post-treatment [19,20]. However, there is no conclusive scientific evidence for the effectiveness of mechanical traction. Therefore, there is a clear need for an RCT to compare mechanical traction to regular treatment options, such as splint therapy, steroid injections, and CTR. Improvements in functional status and symptom severity will be measured using the BCTQ. This questionnaire has been proven to be a valid and reliable outcome measure [21,22]. It is widely used in clinical practice to evaluate the recovery of CTS symptoms after treatment. This means that we have an easy, user-friendly, and quick measure to assess clinical outcome. One strength of this study is the additional outcome measures. Apart from clinical measures, quality of life, absenteeism from work, and health related resource utilization will be measured. Another strength of this study is the follow-up length. Many studies only include a few weeks or months follow-up, while the current study aims at a 12 months follow-up period. A possible limitation of this study is the heterogeneity of the care as usual group. The patients in this group can receive different forms of treatment or no treatment at all. However, since this represents the general practice in an outpatient neurology clinic, the results of this RCT will provide clinically relevant information.

Since the current treatment options are not effective in all patients, or only short-term, research into an alternative, long-term effective treatment option will be of great clinical value. The proposed RCT will provide possible evidence for mechanical traction as a new non-invasive treatment for CTS. Since 4% of the general population develops CTS, the results of this trial will be of benefit to a large patient population. Surgery is often considered a last resort and many patients postpone it for as long as possible, resulting in irreversible nerve damage in some cases. Furthermore, contrary to CTR, mechanical traction does not interfere with work or other daily activities. A less invasive treatment may also have a positive influence on quality of life. Therefore, mechanical traction provides a safe treatment option as an alternative to usual care.

## Trial status

Inclusion has started October 2013. Inclusion is estimated to take up to a year, with one year follow-up. Final results are expected end of 2015.

## Abbreviations

BCTQ: Boston Carpal Tunnel Questionnaire; BMI: body mass index; CTR: carpal tunnel release; CTS: carpal tunnel syndrome; EMG: electromyogram; FSS: Functional Severity Scale; PHQ-4: Patient Health Questionnaire-4; RCT: randomized controlled trial; SSS: Symptom Severity Scale; WHOQOL-BREF: World Health Organization Quality of Life - abbreviated version.

## Competing interests

The authors declare that they have no competing interests.

## Authors’ contributions

MM participated in the trial design and drafted the manuscript. BO participated in the trial design and helped draft the manuscript. BM participated in the trial design and critically revised the manuscript. VP created the trial design and helped draft the manuscript. All authors read and approved the final manuscript.
